# Comparison of electromagnetic and optical navigation assisted Endo-TLIF in the treatment of lumbar spondylolisthesis

**DOI:** 10.1186/s12891-022-05443-1

**Published:** 2022-06-01

**Authors:** De-rong Xu, Liang-rui Luan, Xue-xiao Ma, Zhi-chao Cong, Chuan-li Zhou

**Affiliations:** 1 Department of Spine Surgery, The Affiliated Hospital of Qingdao University, Qingdao, 266000 Shandong Province China; 2 Hi-Tech Zone Li Min Hospital of Weihai Central Hospital Medical Group, Weihai, Shandong Province People’s Republic of China 264209

**Keywords:** Endo-TLIF, Electromagnetic navigation, Optical navigation

## Abstract

Uniportal full endoscopic posterolateral transforaminal lumbar interbody fusion (Endo-TLIF) with percutaneous pedicle screw fixation is a promising, minimally invasive method for the treatment of lumbar spondylolisthesis. However, repeated radiation exposure from X-rays and the steep learning curve remain to be improved.

## Introduction

Low back pain is a common clinical symptom in patients with spinal surgery. Among which, lumbar spondylolisthesis contributes as one of the major cause. When comorbid with lumbar instability and lateral recess stenosis, lumbar spondylolisthesis may further induce lower limb pain and even walking disabilities, severely affecting the patients’ quality of life. Traditionally, the most popular treatment trend has been toward pedicle screw and lumbar interbody cage placement with spinal fusion. Disruption of the normal muscular and bony architecture in the traditional surgery which requires extensive paraspinal decollement, facetectomy and laminectomy would lead to massive bleeding, epidural scar conglutination as well as other potential complications [1, 2]. On the contrary, the recent development of minimal invasive techniques in treatment of spondylolisthesis with applications of spinal endoscopy would ideally assist in reducing the above mentioned complications to a great extent [3, 4]. Different approaches using the full endoscopic techniques, such as interlaminar ipsilateral and contralateral approach, allow the surgeon to perform in the central, lateral recess, and extraforaminal region when treating spondylolisthesis [5]. According to literature, endoscopic lumbar decompression with percutaneous pedicle screw fixation can achieve good clinical results in spondylolisthesis treatment [6, 7].

One of the major technical pearl in uniportal full endoscopic transforaminal lumbar interbody fusion (Endo-TLIF) lies in the ideal positioning of pedicle screw placement [8]. Conventionally, this is achieved by fluoroscopy guidance in the lateral or anterior-to-posterior projection. However, fluoroscopy is relatively technical demanding which relies on the surgeons’ personal skills and experience. Furthermore, it would increase operative time and exopsure to ionizing radiation due repeatedly fluoroscopy. Unfortunately, a high rate of malposition still exist [9]. Additionally, anatomical structures under endoscopic views are rather perplexing and can therefore pose a great challenge to novices. As a result, it may increase operative time and intraoperative radiation exposure as well as carry the risk of vascular and nerve injury [10]. Therefore, it is imperative to find a modified technology that would assist the surgeons to place pedicle screws more accurately and locate and identify the endoscopic anatomy more easily.

Navigation systems has been widely used in surgeries especially in those that requires accurate positioning by demonstrating information concerning the anatomy and guiding the surgical orientation in real time [11]. In spinal surgeries, there are primarily two most commonly used systems: optical navigation and electromagnetic navigation systems [12]. Originally, most optical navigation systems were developed based on infrared LEDs tracking. To start with, preoperative image and anatomy were integrated by relevant systems, then, the surgical procedure was tracked via LEDs light that was captured by the receiving cameras <Shurkhay>. However, theses devices were usually bulky and heavy. More importantly, the sight of LEDs could not be hindered during navigation thus restricting the conventional range of movement of the surgeon with poor handling [13].

In comparison, the electromagnetic navigation system is more flexible due to minimal requirement of the operating dimensions owning to the internal reference electrodes within the instruments. Moreover, this kind of device is free from the line-of-sight limitations met in optical navigation system because of the ability of electromagetic field to penetrate body. Therefore, electromagnetic navigation is more workflow-friendly for its use in spinal minimally invasive and percutaneous procedures [14].

In this study, Endo-TLIF procedures were assisted by either electromagnetic or optical navigation systems. The purpose of this study was to present clinical outcomes of the electromagnetic navigation system and optical navigation system based on our data analysis.

## Data and methods

### Data

The presenting study had been approved by the ethics committe of Affiliated Hospital of Qingdao University and all patients have signed informed consent. From May 2019 to November 2020, we have collected data of patients who received electromagnetic and optical navigation–assisted Endo-TLIF with percutaneous pedicle screw fixation due to single-segment lumbar spondylolisthesis. All patients were presented with persisting lower back pain or claudication and failed standard conservative treatment of at least 3 months. The patients were chosen to receive surgical intervention due to following conditions: (1) Degenerative spondylolisthesis of one segement with sloping of grade 2 or below; (2) when comorbid with bilateral isthmic spondylolisthesis; (3) X-ray that suggests lumbar instability (sagittal translation of segmental vertebral 4 mm or 8% and a sagittal rotation 10 in L1 to L5 and 20 in L5 to S1); (4) neurogenic claudication due to unilateral lateral recess stenosis. Exclusion criteria were as follows: history of (1) spinal fusion surgery, (2) vertebral fracture, (3) spinal tumor, (3) infection, (4) spinal deformity with cobb’s angle over 20-degree on the coronal plane. Visual analog scale (VAS) and Oswestry Disability Index (ODI) scores for lower back pain and limb pain were used at preoperative and follow-up examinations. The modified MacNab standard was used to evaluate early postoperative efficacy. Spinal 3D reconstruction were reconstructed using preoperative computed tomography (CT) scan data with slice thickness of 1 mm. Patient’s demographic such as age, gender, classification of spondylolisthesis and radiological image were recorded for baseline comparison.

### Methods

The Ethics Committee of Affiliated Hospital of Qingdao University approved the study, and all patients signed informed consent. The methods described were performed in accordance with relevant guidelines and regulations. This was not a study commissioned or funded by any manufacturer.

This retrospective study explored the effects of electromagnetic navigation (group A) and optical navigation (group B) on the outcomes of Endo-TLIF. Before using the two types of navigation, surgeons have received systematic training and can skillfully use the two navigation systems. The operation of the system is not affected by the operation time and different surgeons. The operative blood loss, rate of adjustment for guide wires, frequency of X-ray exposure, operative time, accuracy of pedicle screw placement, and clinical outcomes were recorded. The clinical outcomes in both groups were evaluated using visual analogue scale (VAS) and the Oswestry Disability Index (ODI). The two techniques were compared according to the results.

### Surgical procedures of electromagnetic navigation–assisted Endo-TLIF (group a)

#### Preparation of electromagnetic navigation

**Fig. 1:**
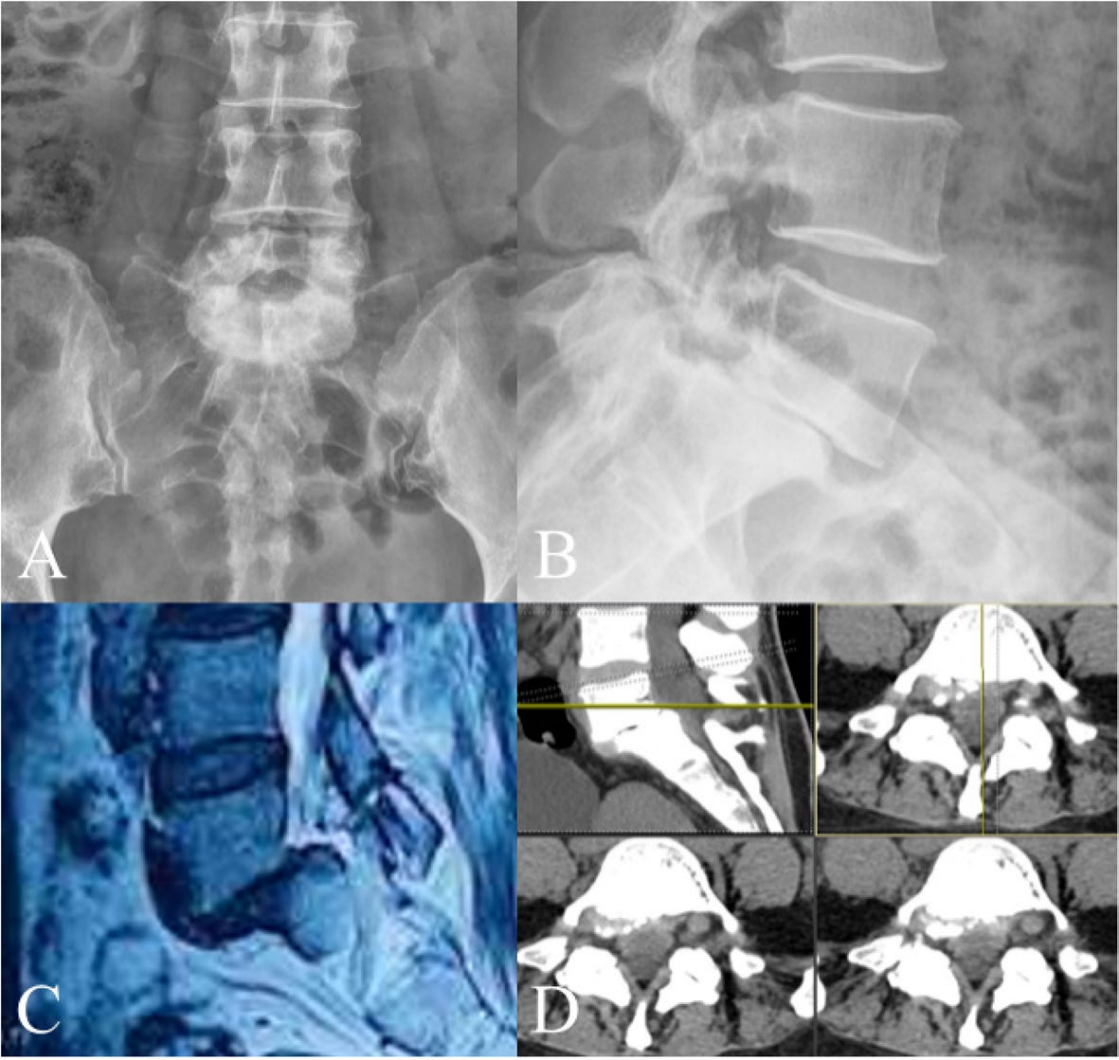
Representative case of a patient before Endo-TLIF. **A** and **B**: the fluoroscopic AP (**a**) and lateral (**b**) views. **C** and **D**: CT scan and MRI of the case with lumbar spondylolisthesis

**Fig. 2:**
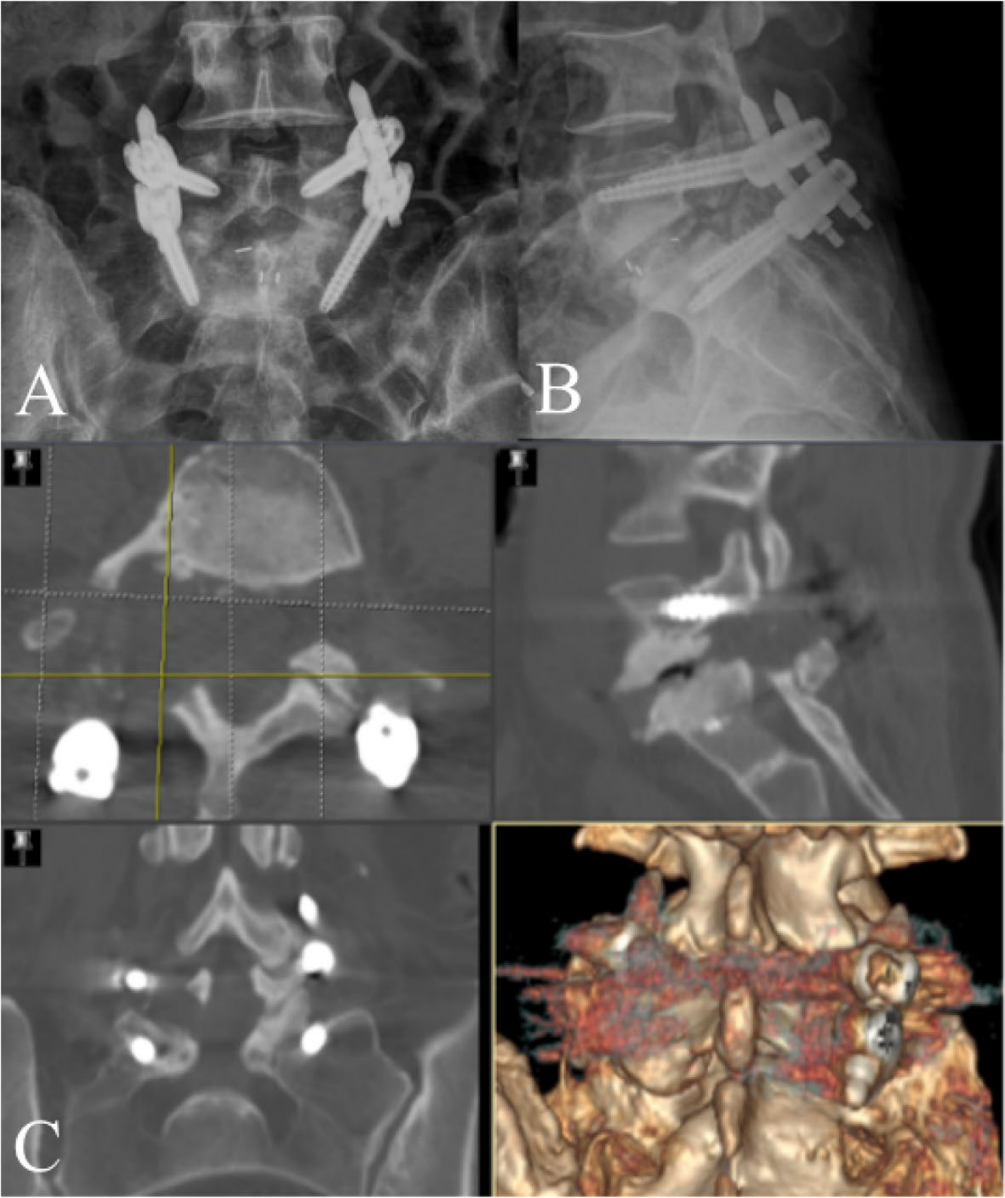
**A** The result of transcranial electrical stimulation-induced motor-evoked potentials (MEPs) and electromyography (EMG) monitoring during the operation. **B** The surgical incision. **C** An interbody fusion cage was observed under endoscope

**Fig. 3:**
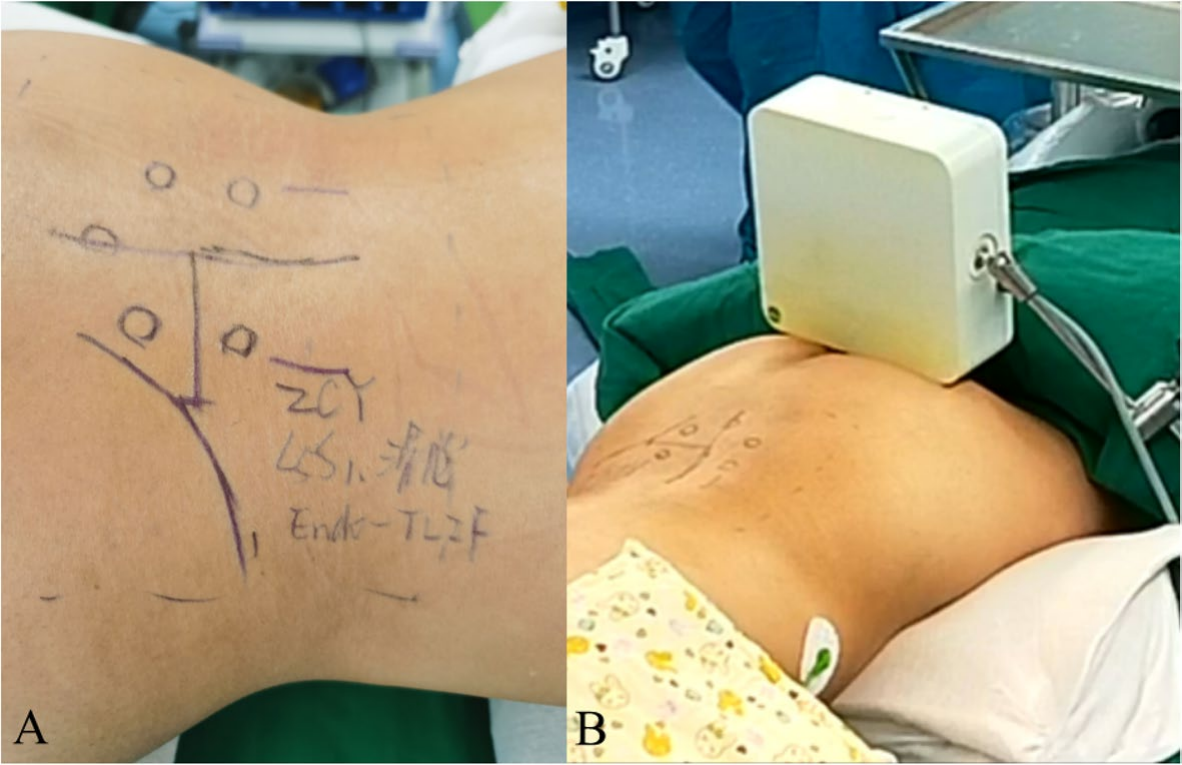
The incision plan. **A** According to C-arm positioning, bilateral iliac crest and pedicles were marked. **B** Navigation sensor frame is fixed beside operating bed

In order to demonstrate the procedure more clearly, we have herein taken L5/S1 segment as an example **(**Fig. 1**)**. Patients were placed in the prone position with spine slightly flexed under general anesthesia. All patients underwent transcranial electrical stimulation-induced motor-evoked potentials (MEPs) and electromyography (EMG) monitoring during the surgery **(**Fig. 2**)**. After C-arm positioning, bilateral pedicles of L5 and S1 were crudely marked by the surgeon. **(**Fig. 3**)**.

First, an electromagnetic field must be generated by fixing the field generator on the patients’ surface **(**Fig. 3**)**. Then, the device embedded in that electromagnetic field is detected with signal coils. Next, we placed a reference coil (patient tracker) on the spinous process or the ilium. In this way, we were able to map the generated intraoperative electromagnetic field with the preoperative 3-D image based on CT scan. The devices and instruments used were as follows: a Center Pointer, Access Tracker, Spine Pointer and a matched navigable screwdriver. We used the Center Pointer in the process of surface matching and pedicle drilling. The Access Tracker acted as a measuring instrument to evaluate the screw length entering the vertebral body at a specified direction. The Spine Pointer acted as a safety instrument that could detect screw malposition or surrounding cortical bone destruction. All instruments mentioned above interacted with the navigation system via wire connections.

**Fig. 4:**
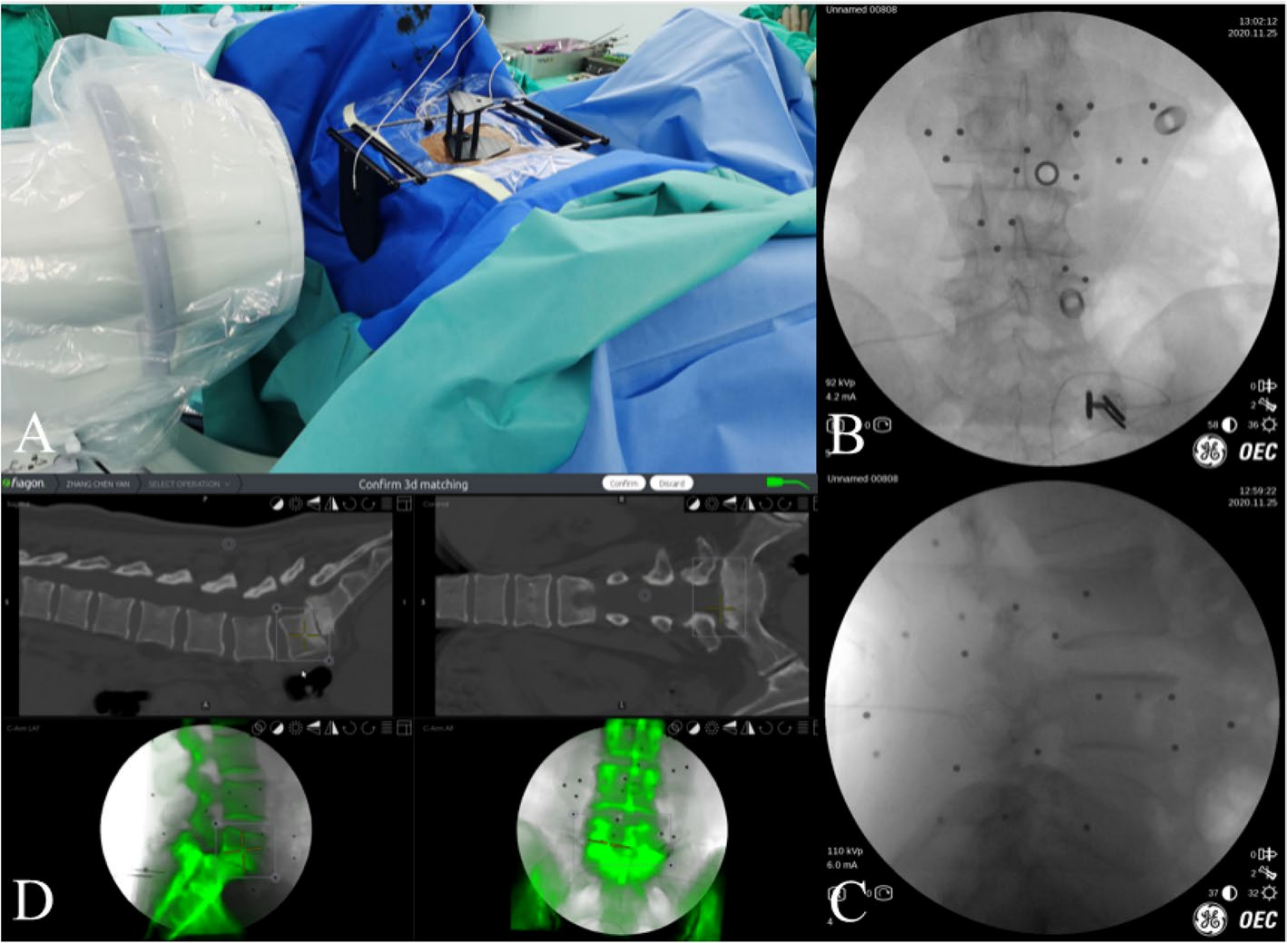
Preparation of Electromagnetic navigation. **A** After the field generator and a patient tracker equipped with signal coils was fixed on the ilium. **B** and **C** Anteroposterior and lateral fluoroscopic views of the lumbar segment were taken. **D** The software made surface matching on the respective vertebral body with the preoperative CT data in the electromagnetic coordinate system

After finishing instruments preparation, we performed surface matching by taking anteroposterior and lateral fluoroscopic graphs of the targeting segments into the navigation system to match with the preoperative CT based image **(**Fig. 4**)**. Finally, optical check on anatomical landmarks was performed following systemic self-correction. The whole time for the navigation system to prepare took approximately 10–15 min.

#### Insertion of guide wire

**Fig. 5:**
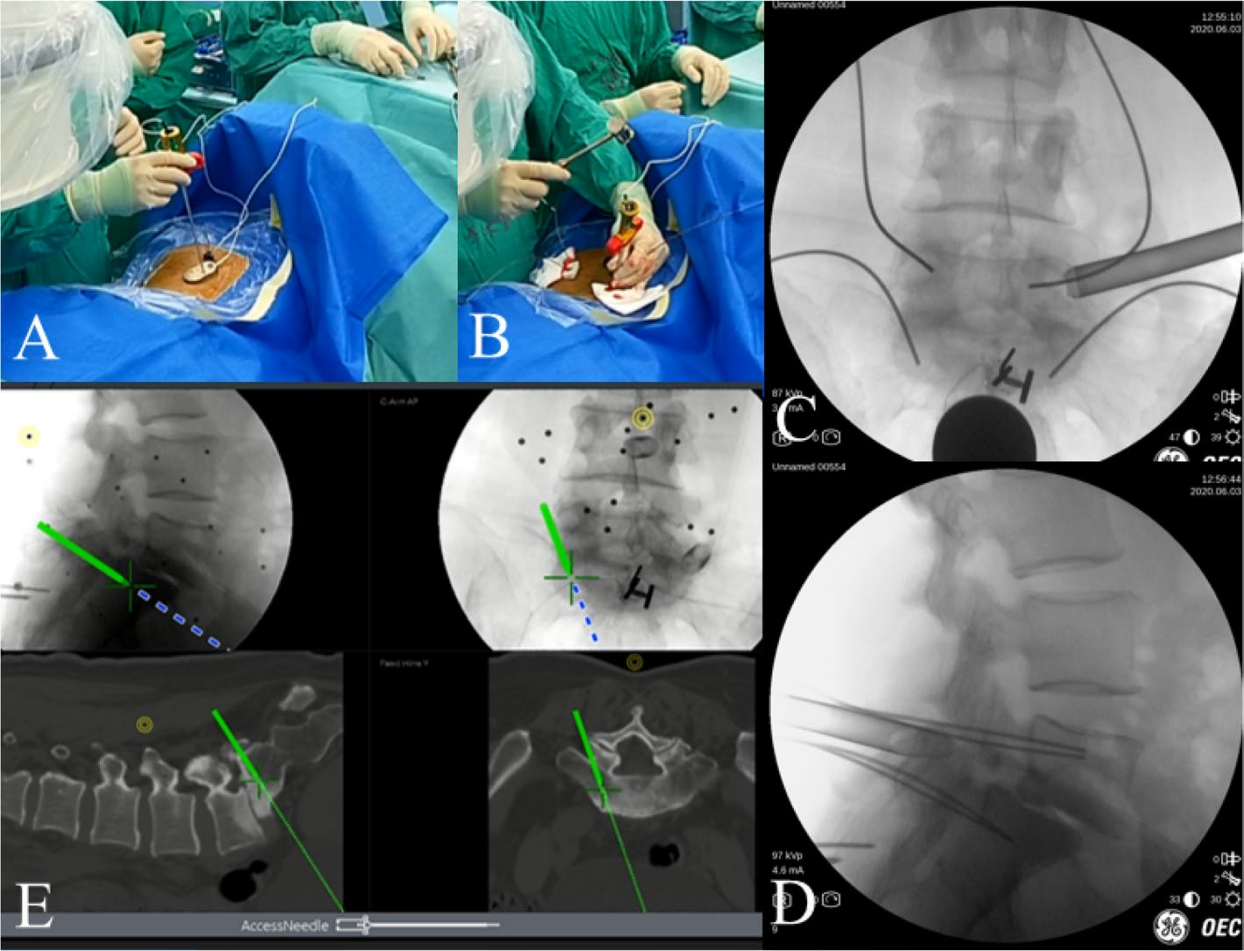
**A** Access Tracker was successfully registered. **B** and **E** Guide wires insertion. In the navigation system, the procedure and track of Access Tracker is real-time visible in all spatial planes so the operator can make a quick adjustment as needed. **C** and **D** Determine the position of the guide wire and the endoscopic working channel with anteroposterior and lateral fluoroscopic views

Based on the precise position of the pedicles screw insertion, the incisions were made. By using the Access Tracker, it was made possible to visualize the surgical procedures in all spatial planes in a timely manner so that the surgeon could make dynamic adjustment when necessary. A pilot hole was made after drilling with assistance of Access Tracker. Then, after pedicle opening, a guide wire (300 mm × 1.5 mm) was inserted under accurate guidance of the navigation system **(**Fig. 5**)**.

#### Decompression and cage placement under endoscope

**Fig. 6:**
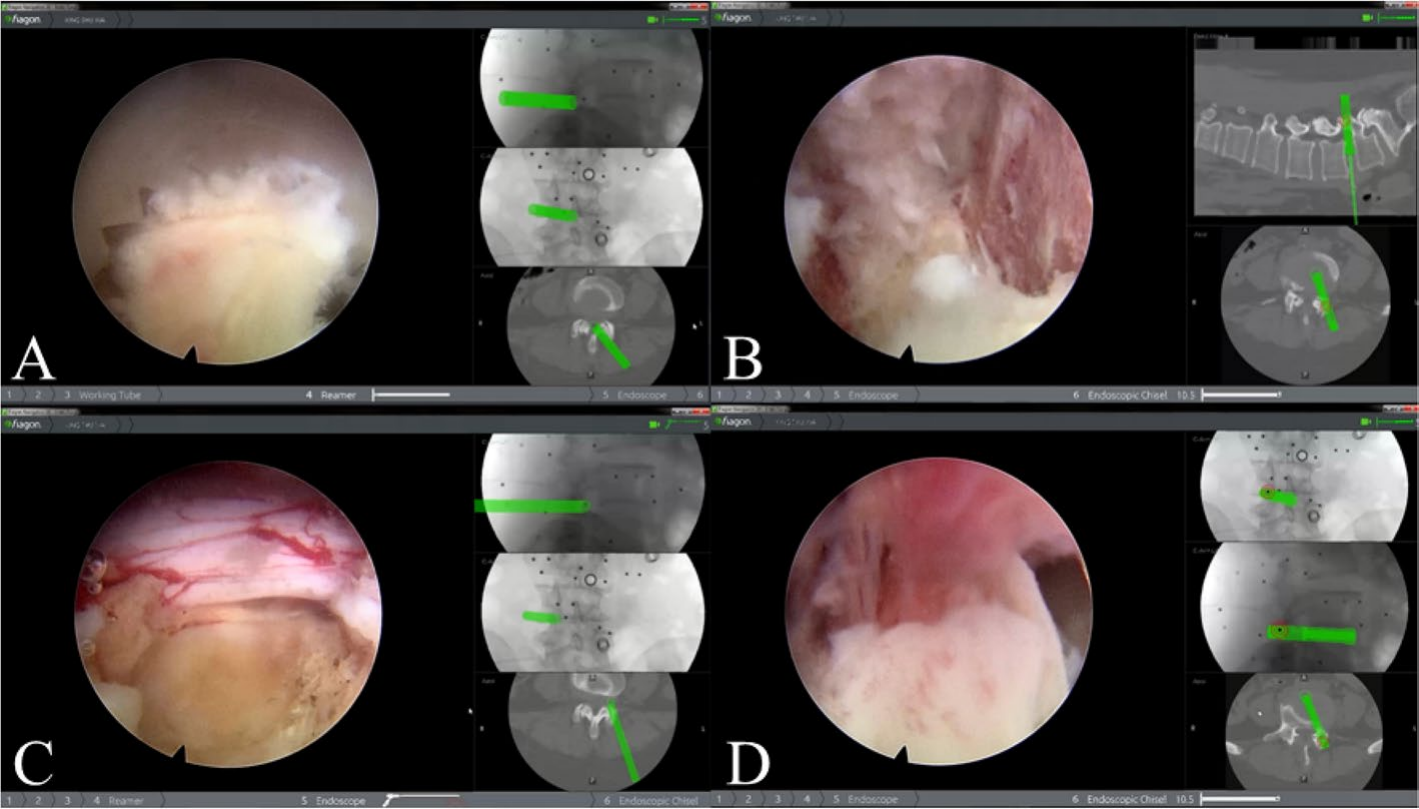
Operation field under endoscope, green indicates safe operation. **A** The Laminotomy was achieved via the circular saw assisted by electromagnetic navigation under the view of the endoscope. **B** The discectomy was achieved assisted by electromagnetic navigation under the view of the endoscope. Cartilage endplate was exposed. **C** The dural sac was exposed, and pulsation of the dural sac improved. **D** The model cage was implanted to the center of the intervertebral space in appropriate depth, with the location confirmed by electromagnetic navigation

Endo-Surgi PLUS endoscopic system (Joimax Inc.) was used for the following procedure. First we inserted the endoscopic cannula on the undersurface of the superior face. Then, a uniportal endoscope was inserted after tissue dilation and removing the cannula expansion. The following anatomical landmarks were able to be visualized directly under direct endoscopy: the S1 superior facet joint, L5 inferior facet joint tip, the isthmus, and part of the vertebral plate. Additionally, they were shown and verified by Access Tracker from the navigation system. Next, the foramen was enlarged by using a circular saw to remove part of the superior facet joint of the S1 and inferior facet joint tip of L5 **(**Fig. 6**)**. A working cannula was created after exposure of ligamentum flavum and protection of the dural sac and S1 nerve root for the next step of spinal canal decompression **(**Fig. 6**)**.

With the assistance of Access Tracker, it was made possible for the surgeon to identify the location as well as direction of the intervertebral disc. The degenerated nuclear material was removed using reamers of different diameters through the working cannula. After an endplate preparation was finished, the Access Tracker was able to reach the end of the intervertebral space to evaluate the processed depth **(**Fig. 6**)**.

**Fig. 7:**
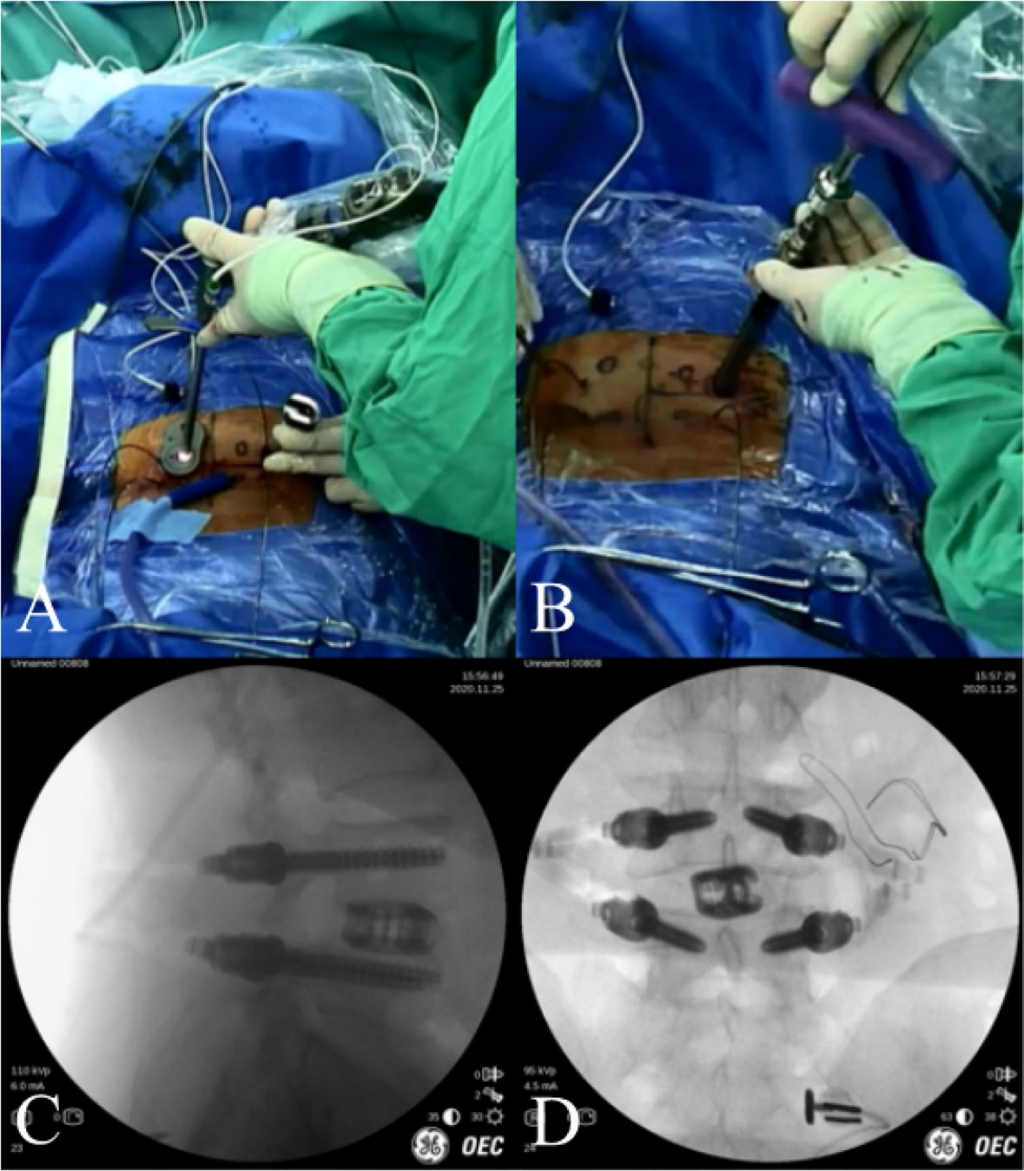
**A** The circular saw was registered to be connected to the electromagnetic navigation system. **B** The pedicle screws were installed to replace four guide wires. **C** and **D** The position of screws and cage were verified under C-arm fluoroscopy

Location of the model cage which was placed at the center of intervertebral space at an appropriate depth was confirmed by intraoperative fluoroscopy on both anteroposterior and lateral views **(**Fig. 6**)**. After cage model removal, we filled the intervertebral space with autograft and allograft. Then, we were able to restore lumbar lordosis as well as the height of intervertebral space by inserting cage through the working cannula. Finally, four cannulated pedicle screws were inserted into the pedicle channel via the guide wires. After guide wires removal, we used C-arm fluoroscopy to confirm the position of cage and pedicle screws placement **(**Fig. 7**)**.

### Surgical procedures of optical navigation–assisted Endo-TLIF (group 2)

#### Preparation of optical navigation

We also took L5/S1 segment as an example. All procedures were performed in the prone position under general anesthesia with the spine held in slight flexion. Patients received transcranial electrical stimulation-induced motor-evoked potentials (MEPs) and electromyography (EMG) monitoring during the operation. We used the O-arm (Medtronic, Inc.) to obtain AP and lateral plain radiographs to mark the bilateral pedicles of L5 and S1 roughly. Images were viewed on the StealthStation navigation system (StealthStation S7, Medtronic Inc.), which was positioned adjacent to the patient’s caudal site.

The surgical area was disinfected and covered with a sterile towel. The optical navigation reference frame (patient tracker) was fixed to the patient’s sacrum. A full O-arm spin was then performed to generate CT images (the tracker need to be included in the view). Then, the CT data were uploaded to the optical navigation workstation and surgical instruments which may be used were prepared after registration.

#### Guide wire insertion

**Fig. 8:**
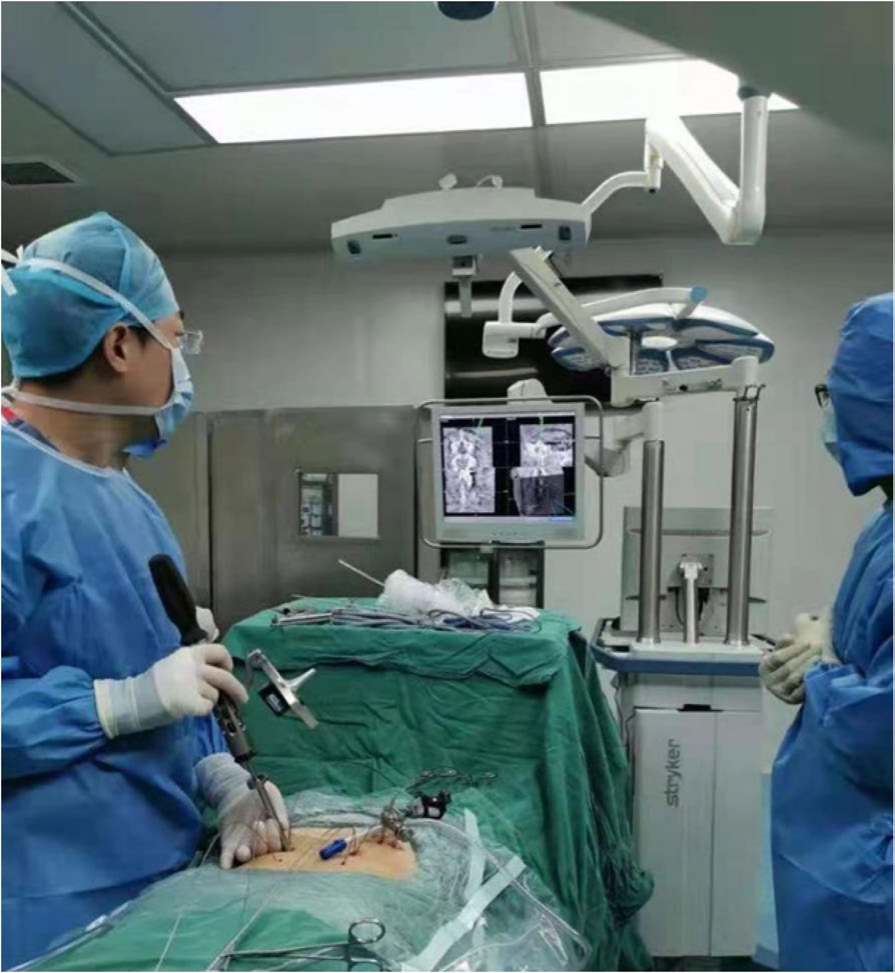
A case of Endo-TLIF assisted by optical navigation navigation. **A** and **B** Intraoperative C-arm image of the guide wires. **B** and **C** The position of screws and cage were verified under C-arm fluoroscopy

The incision was designed according to the precise position of the pedicles in the navigation system. The drill, which was registered to the Stealth system, was used to drill a pilot hole. Then, the surgeon continued to open through the pedicle and inserted a guide wire (300 mm × 1.5 mm) under accurate navigated guidance **(**Fig. 8).

### Decompression and cage placement under endoscope

**Fig. 9:**
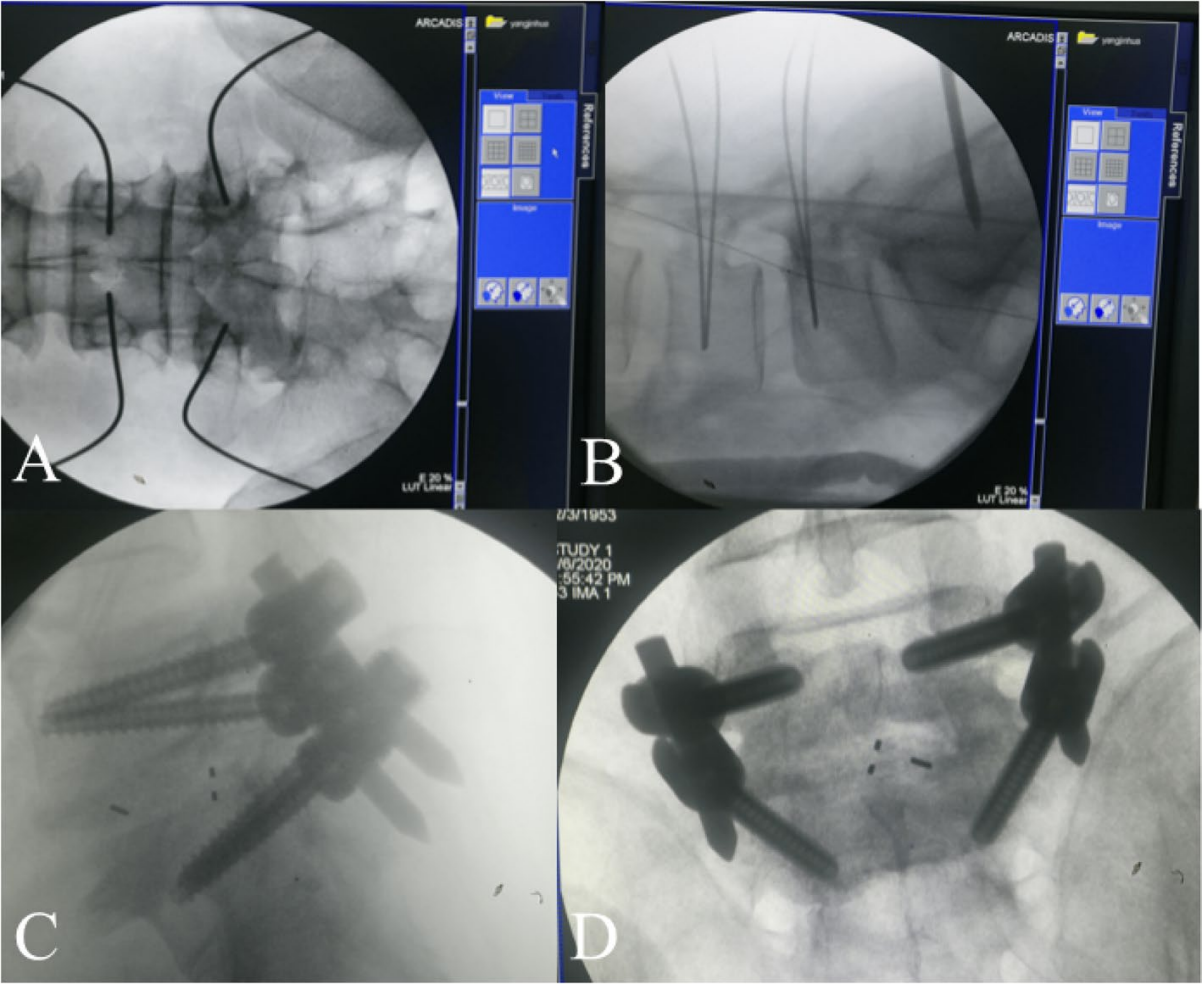
The pedicle screws were installed assisted by optical navigation

This procedure was the same as the electromagnetic navigation group except that there was no navigation aid **(**Fig. 9**)**.

### Closure

C-arm fluoroscopy was used to confirm the optimal position after insertion of the guide wires into the vertebral pedicles in both groups. Finally, the incisions were sutured to close the wound. We have also recorded the frequency of adjustment, ie, times of guide wires corrections needed until a satisfactory pedicle screw was placed. The surgical field was closed without drainage placement. Postoperative mangement 48 hours following the surgery included dehydration, analgesia, anti-inflammatory, and neurotrophic treatment.

**Fig. 10:**
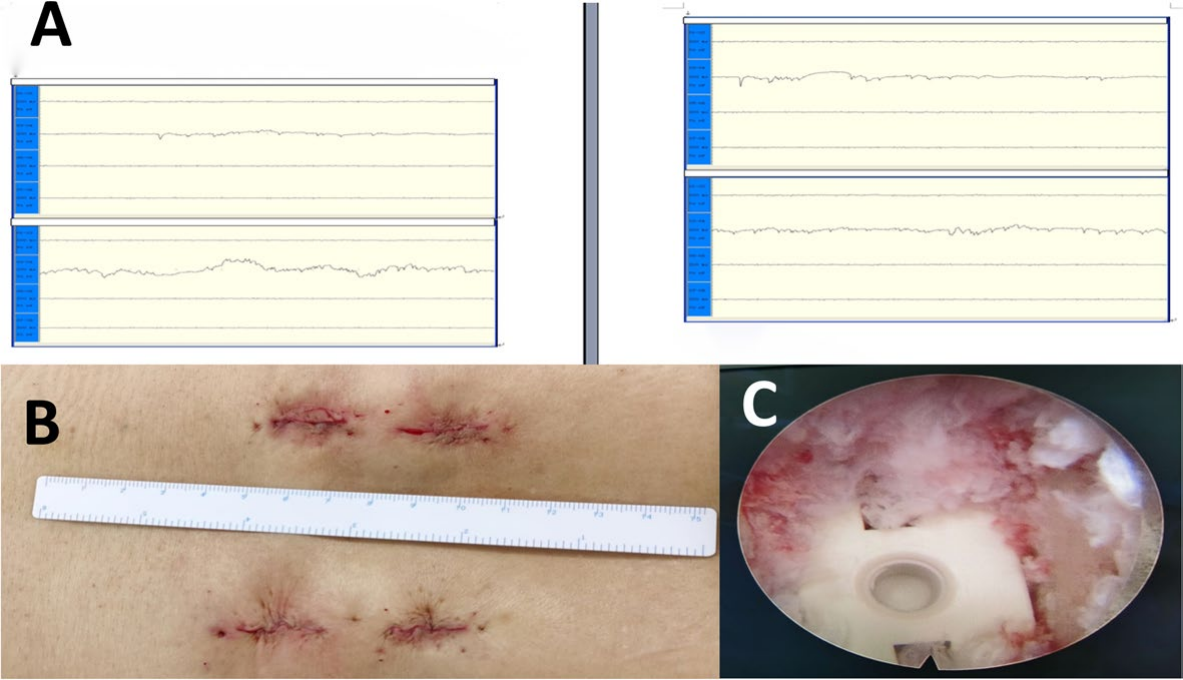
Postoperative radiographs (**A** and **B**) and CT scan (**C**)

The distance between the screw surface and pedicle cortical wall in the perpendicular direction was assessed based on postoperative CT scan with 3D reconstruction **(**Fig. 10**)**. The screw location was then subdivided into four grades depending on the pedicle screw positions as described in the previous literature [15] **(**Table 1**)**. Excellent and good position were represented by grade 0 and grade 1, respectively. The following data were recorded: operation time (This includes the time required for navigation settings and registration), amount of bleeding, the rate of adjustment for guide wires, amount of X-ray exposures, accuracy of pedicle screw placement, clinical outcomes and hospitalization duration.

**Table 1:** Summary of computed tomography grading criteria

Grade	Accuracy of PPSP according to Neo et al.
0	No deviation; the screw was contained in the pedicle
1	Deviation < 2 mm (i.e., less than half of the screw diameter)
2	Deviation > 2 and < 4 mm
3	Deviation > 4 mm (i.e., complete deviation)

### Statistical analysis

Data analysis were conducted using SPSS statistical analysis software (version 18). Descriptive statistics were used for all variables, and they were shown as mean and standard deviation (SD). Fisher’s exact test was used to analyze categorical variables. After verifying normal distribution, a paired t-test was used to compare preoperative and postoperative VAS scores and ODI scores. A value of *P* < 0.05 was considered statistically significant.

## Results

There were no significant differences between the two groups in gender, age, follow-up time and other baseline data (*P* > 0.05) **(**Table 2**)**. The duration of guide wires insertion,decompression, cage placement and operative time of group A were less than group B and the differences were statistically significant. The pedicle grading of patients between two groups were no significant differences **(**Table 3**)**. The frequency of adjustment for guide wires in group A was less than group B, although there was no statistically significant difference between the two groups **(**Table 4**)**.

**Table 2:** Basic information of the patients

	Electromagnetic navigation group	Optical navigation group	*P*
N	42	46	–
Age (years)	51.83 ± 14.80	56.33 ± 11.04	0.108
Gender (Female/Male)	22/20	25/21	0.683
Surgical segment	–	–	0.578
L3-L4	2	4	
L4-L5	27	25	
L5-S1	13	17	
Etiological Classification	–	–	–
Isthmic	9	14	0.337
Degenerative	33	32	
Meyerding classification	–	–	–
I°	35	40	0.632
II°	7	6	
The follow-up time (months)	6	6	–

**Table 3:** Pedicle grading of patients

	Electromagnetic navigation group	Optical navigation group	*P*
Screws	168	184	0.938
Grade 0	140	150	
Grade 1	22	25	
Grade 2	4	6	
Grade 3	2	3	
Excellent and good rate (%)	96.4%	95.1%	

Grade 0 and Grade 1 was considered as excellent and good position (%).

**Table 4:** Operative data of 2 groups

	Electromagnetic navigation group	Optical navigation group	*P*
Number of screws	168	184	–
Duration of guide wires insertion (min)	15.41 ± 2.68	20.20 ± 3.41	0.000
Duration of decompression and cage placement (min)	73.43 ± 7.04	85.83 ± 7.74	0.000
Frequency of adjustment for guide wires (times)	3	7	0.414
Rate of adjustment (%)	1.78	3.80	
operative time (min)	170.38 ± 14.79	180.74 ± 14.84	0.002
Operative blood loss (ml)	20.45 ± 5.52	20.04 ± 5.59	0.731
Frequency of X-ray exposure (times)	8.95 ± 1.67	8.54 ± 1.53	0.234
Hospitalization after operation (days)	3.50 ± 0.94	3.24 ± 0.64	0.137

Rate of adjustment = (Frequency/Number of screws).

The postoperative VAS and ODI in 2 groups were significantly lower than those before surgery and the difference was statistically significant(P < 0.05). There were no statistically significant differences in VAS and ODI between the two groups both before and after surgery (P > 0.05) **(**Table 5**)**.

**Table 5:** Clinical outcomes of the patients

	Electromagnetic navigation group	Optical navigation group	*P*
VAS			
Preoperative	8.21 ± 1.09	8.44 ± 1.00	0.327
Last follow up	1.19 ± 1.09	1.15 ± 1.19	0.876
P	0.000	0.000	
ODI			
Preoperative	81.89 ± 10.60%	82.24 ± 9.60%	0.869
Last follow up	11.48 ± 9.83%	11.90 ± 8.80%	0.832
P	0.000	0.000	

## Discussion

Spinal endoscopy has become more popular in recent years among surgeons and patients because of its advantages such as reduced trauma, less bleeding and quick recovery in the treatment of lumbar degenerative diseases. In addition to disc herniation and spinal stenosis, patients with lumbar instability and spondylolisthesis can now also receive better treatment and obtain good clinical outcomes through spinal endoscopic lumbar fusion surgery. Wu Wenjie et al. [16] compared the efficacy of Endo-TLIF and MIS-TLIF in the treatment of lumbar degenerative diseases and found that both types of surgery could achieve the same effect. However, Endo-TLIF has the advantages of smaller injury, quicker recovery, lower cost, and better development prospects, therefore, it is worthy of superior recommendation. Pang Hung Wu, et al. revealed that uniportal endoscopic posterolateral lumbar interbody fusion could also be applied in the treatment of severe foraminal stenosis secondary to severe collapsed disc space. According to their results, disc height were able to be restored significantly and VAS score decreased 4.3 ± 1.0 at the last follow-up [17]. In Endo-TLIF surgery, there were several technical steps, including percutaneous pedicle screw insertion, spinal decompression, discectomy and intervertebral bone graft and fusion device placement. Approaches for percutaneous pedicle screw placement include placement by hand, under fluoroscopic guidance, with 3D fluoroscopy and intraoperative CT/MRI guidance, etc. Studies have reported that the failure rate of screw placement could be as high as 20–40% [18, 19]. Moreover, failure of screw placement is often associated with spinal cord and blood vessel injury [20, 21], which requires revision and screw readjustment. In addition, the traditional screw placement technology requires high clinical experience and well basic knowledge reserve from the surgeons. The learning curve of pedicle screw placement technology is long,ie, it takes a long time to train an orthopaedic surgeon who can independently complete the operation. Moreover, in order to ensure the safety and accuracy of pedicle screw placement, repeated fluoroscopy is needed to adjust the entry point and trajectory direction of the screw path, which not only increases the radiation exposure of all medical staff and patients involved in the operation [22], but also prolongs the operation time and reduces the efficiency of the operation. Therefore, how to efficiently and accurately place the screw, shorten the operation time and reduce the risks has become a problem that we need to pay attention to.

In order to reduce intraoperative radiation exposure to improve the safety of operation and shorten the operation time, a variety of computer assisted navigation systems (CANS) have been developed. At present, optical navigation and electromagnetic navigation are the most commonly used ones in clinical practice. The optical navigation system through O arm access to match on time in the operation space coordinates, and put it with preoperative abstracted to obtain images of the point of registration [23]. After registration, the surgery can be visualized in real-time in terms of surgical instruments,internal fixation device and spinal structure as well as their spatial relations, thus reducing the amount of intraoperative fluoroscopy. The real-time visualization can prevent bone cortex being broken through by the surgical instruments or internal fixation and the consequent injury of peripheral nerves and blood vessels. In addition, through real-time intraoperative imaging guidance, the learning curve of pedicle screw placement can be shortened, so that young physicians can master pedicle screw technology faster. The advantages of optical navigation-assisted surgery are as follows: 1. It effectively reduces the amount of intraoperative fluoroscopy and reduces the risk of radiation exposure to doctors and patients [24]; 2. The operation time can be efficiently shortened by reducing the dependence of C-arm during the operation [25]. 3. The track of the screw path can be monitored in real time which allows prompt adjustments to reduce the probability of misplacement when placing pedicle screws; 4. It can effectively assist young doctors to perform surgery and shorten the learning curve; 5. Assist planning of surgical incisions to reduce trauma and intraoperative bleeding. However, optical navigation also has some problems: 1. When different instruments are used, they need to be replaced and the system requires reselection of the instruments and re-registration, which increases the operation time to a certain extent; 2. When tracking light is blocked during the operation, the system interface will be frozen and the location of the nail path cannot be reflected in real time, which can be misleading to the operator; 3. Due to the influence of many factors, optical navigation can only assist pedicle screw placement but cannot assist the steps of spinal decompression, intervertebral disc handling and lumbar fusion device placement under the microscope. Jin et al. [26] performed surgery with the C-arm assistance using the Uniportal Endoscopic System, with an average operative time of 213.8 ± 31.7 min. According to Heo et al. [6].,the mean estimated blood loss was 85.5 ± 19.41 mL. In this paper, the mean operative time and blood loss of the Optical navigation group were 180.74 ± 14.84 min and 20.04 ± 5.59 mL, respectively. Therefore, the use of optical navigation can shorten the operative time and reduce intraoperative blood loss. The average number of fluoroscopic procedures was 8.54 ± 1.53, which was a significant improvement when compared with the procedure without optical navigation assistance [27].

The electromagnetic navigation equipment in this study can perfectly overcome some problems existing in optical navigation. Based on the preoperative CT scan data, the electromagnetic navigation system matches the actual position of the spine in the electromagnetic field with the preoperative CT 3D reconstruction model and displays the relative positions of the internal fixation device, surgical instruments and the spinal structures in the surgical area in real time on the electromagnetic navigation system. It not only possesses the advantages of optical navigation such as reduced ray exposure, operation time saving, real-time monitoring of screw track, shortened learning curve and assistance of planning surgical incision but also overcomes some of the problems existing in optical navigation: 1. Electromagnetic navigation relies on the magnetic field emitter for spatial positioning, without having to worry about light blocking; 2. If the device needs to be replaced during the operation, just insert the guide wire into the new device and register it again. The magnetic navigation system can automatically identify the new device without any need to adjust the system; 3. Electromagnetic navigation can not only assist the placement of pedicle screws, but also match the relevant instruments for the treatment of vertebral space with reamer and assist the completion of the steps under the microscope thus achiving the real-time assistance of electromagnetic navigation throughout the whole process. Von Jako et al. [28] made a comparison between electromagnetic navigation and traditional C-arm assisted screw placement and found that electromagnetic navigation assisted pedicle screw placement had a higher accuracy. In this study, according to preoperative and postoperative VAS and ODI, both magnetic navigation and optical navigation could achieve satisfactory surgical results. However, in the electromagnetic navigation group, duration of spinal decompression and discectomy with pedicle screw placement and interbody fusion device placement were significantly shorter and the total operative time was significantly shorter. Compared with optical navigation group, electromagnetic navigation group did not show a significant advantage in reducing intraoperative bleeding, X-ray exposure and hospital stay. Although there was no statistically significant difference in the frequency of adjustment for guide wires between the two groups, the rate of adjustment was significantly reduced by electromagnetic navigation assistance in this study.

Minimally invasive transforaminal endoscopic decompression is a technical-demanding approach with a steep learning curve. It may poise extensive challenges to even experienced surgeons due to complicated anatomical structures under the endoscope, for instance, in scenario like intervertebral collapse, severe hyperosteogeny and revision surgery [29]. Such challenges would hinder the promotion of its clinical applications. By using a single guidance instrument which could show the surgical instruments and their spatial relations with the surrounding structures, the Access Tracker, it was made possible for a clear visualization of this procedure when adopting the electromagenetic navigation system. In addition, when in combination with use of a small noninvasive instrument that allows automatic registration, the Isee Tracker, they would ensure all the benefits of this minimally invasive surgical procedure and shorten the learning curve. Moreover, the Access Tracker serves in assistance of identifying key anatomical structures such as the superior facet joint, inferior facet joint tip, isthmus, and part of the vertebral plate. Access Tracker can also be used in detection of severe collapsed disc prior to removal of the intervertebral disc tissues. Finally, pre-evaluation of the depth and height of the intervertebral space could be made by the Access Tracker before cage implantation. All of these technical pearls mentioned above serve as an irreplaceable improvement to promotion of the safety and efficacy of this challenging procedure when compared with either conventional approaches or even under opitcal navigation assistance.

Although electromagnetic navigation has many advantages, it also has some disadvantages. First, some technical defects were still found during the course of our application. Although not possible to be interfered by opitcal hinderance, the system matched image could sometimes be disturbed from metallic devices such as C-arm. The registration process was still time-consuming due to complicated procedures especially for young surgeons. Therefore, we hope the navigation system can be further modified by simplifying the registration and matching procedure and strengthening anti-interference capability as well as improving reliability with self-diagnostic and correction functions when encountering errors so as to complete the surgery more conveniently and efficiently. Indications for other parts of the spine such as electromagnetic navigation assistance for the cervicothoracic junction should also be developed and expanded.

Our study have the following shortcomings: first, the inherent differences and selection bias of the enrolled cases could not be fully avoided due to its retrospective nature. Second, sample size was relatively small and follow-up time was short, therefore, it could not demonstrate clinical outcomes and complications in the long-term. A multi-center, large sample, prospective, randomized controlled trial with longer follow-ups should to be performed to overcome such shortcomings and provide more solid clinical evidence.

## Conclusion

Comparison of electromagnetic and optical assisted navigation in Endo-TLIF have not been reported in previous literature. Our results suggest that electromagnetic navigation, like optical navigation, is a reliable technique for percutaneous pedicle screw placement with satisfactory clinical outcomes. However, when compared with optical navigation, electromagnetic navigation assisted pedicle screw placement has more advantages in terms of screw placement accuracy and operation time saving. In addition, different from optical electromagnetic navigation, electromagnetic navigation system has been able to play an increasingly important role in the steps of spinal decompression, intervertebral space clearance and interbody fusion cage placement under the microscope.

## Data Availability

The datasets generated and analyzed during the current study are available from the corresponding author on reasonable request.

## Abbreviations

Endo-TLIF: uniportal full endoscopic transforaminal lumbar interbody fusion
VAS: visual analog scale
ODI: Oswestry Disability Index
MEPs: motor-evoked potentials
EMG: electromyography
