# Correction: Comparison of electromagnetic and optical navigation assisted Endo-TLIF in the treatment of lumbar spondylolisthesis

**DOI:** 10.1186/s12891-022-05649-3

**Published:** 2022-07-19

**Authors:** De-rong Xu, Liang-rui Luan, Xue-xiao Ma, Zhi-chao Cong, Chuan-li Zhou

**Affiliations:** 1 Department of Spine Surgery, The Affiliated Hospital of Qingdao University, Qingdao, Shandong Province 266000 China; 2 Hi-Tech Zone Li Min Hospital of Weihai Central Hospital Medical Group, Weihai, Shandong Province 264209 People’s Republic of China

**Correction: BMC Musculoskelet Disord 23, 522 (2022)**

**https://doi.org/10.1186/s12891-022-05443-1**

Following the publication of the original article [1] the authors would like to correct “joimax Inc” to “unintech GmbH” in the first paragraph under the heading *Decompression and cage placement under endoscope*.

The original article [1] has been updated.
